# Pro-Tumorigenic Signaling Between Small Extracellular Vesicles of Cancer Cells and Bone Marrow-Derived Mesenchymal Stem Cells—An In Vitro Study

**DOI:** 10.3390/ijms27062654

**Published:** 2026-03-13

**Authors:** Jyothi Attem, Ram Mukka Raju Jogula, Swathi Kaliki, Geeta K. Vemuganti

**Affiliations:** 1 School of Medical Sciences, Science Complex, University of Hyderabad, Hyderabad 500046, India or 18bmph01@uohyd.ac.in (J.A.); or rmraju.in@gmail.com (R.M.R.J.); 2 L.V Prasad Eye Institute, Hyderabad 500034, India; swathikaliki@lvpei.org; 3 The Operation Eyesight Universal Institute for Eye Cancers, L.V Prasad Eye Institute, Hyderabad 500034, India; 4 Prof. Brien Holden Eye Research Centre, L.V Prasad Eye Institute, Hyderabad 500034, India

**Keywords:** retinoblastoma, extracellular vesicles, metastasis, chemoresistance, stemness

## Abstract

Retinoblastoma (Rb) is an intraocular tumor caused by genetic alterations in the RB1 and MYCN genes within developing retinal cells. Chemoresistance and metastasis are major challenges for treatment, with the bone marrow (BM) representing the most common metastatic site. We investigated the effect of tumor-derived sEVs (TDsEVs) on the crosstalk between metastatic site cells (BM-derived mesenchymal stem cells (BM-MSC)) and tumor cells, and characterized them according to MISEV guidelines. The uptake of sEVs and the associated phenotypic changes in the BM-MSCs were analyzed with confocal microcopy. The functional effects were assessed through MTT assays for viability, scratch and Transwell assays for migration, and colony- and sphere-formation assays to evaluate clonogenicity and self-renewal, while stemness marker expression was examined by immunoblotting. Secretome changes following sEV exposure were analyzed using dot blot assays. sEVs were taken up by both cells. TD-sEVs significantly enhanced BM-MSC migration and induced differentiation into a myofibroblast-like phenotype without affecting cell viability. Conversely, BM-MSC-derived sEVs promoted tumor cell viability, migration, and stemness marker expression. Both the BM-MSCs and tumor cells exhibited altered secretory profiles after sEV treatment. The in vitro findings provide cumulative evidence that sEV-mediated interactions contribute to a tumor-supportive milieu or premetastatic niche at the BM in Rb.

## 1. Introduction

Retinoblastoma (Rb) is the most common intraocular malignancy in children less than 5 years old, with an incidence of approximately 1 in every 15,000–20,000 live births worldwide [1]. This disease arises in developing retinal cells and is primarily driven by biallelic inactivation of the RB1 tumor suppressor (>98% of cases), while a rare subset (~1.5–2%) is RB1-intact and driven by MYCN amplification [2,3,4]. While early-stage Rb is often manageable with treatment, advanced cases (International Classification Groups D and E) and post-treatment recurrence or metastasis after treatment remain significant clinical challenges [5]. Unlike other solid tumors, invasive tumor tissue biopsy is contraindicated in Rb because of the poorly cohesive and loosely attached tumor cells. When disrupted by a needle or surgical instrument, viable tumor cells easily detach, increasing the chance of cell spillage into the extraocular site, further dissemination to vital organs—either to the central nervous system (CNS) via the optic nerve or to bone marrow (BM) through systemic circulation [6,7,8]. These high-risk metastasis cases frequently require enucleation, and in low- or middle-income countries, over 40–60% of affected children die even after treatment [6]. Clinical and experimental evidence has established BM as the most common site for Rb metastasis and therapy resistance [7,8,9,10,11,12]. This preference is driven by BM’s vascular niche, hypoxic environment, and its richness in stromal cell types including mesenchymal stem cells [10,11,13]. Targeting dormant Rb tumor cells is a compelling strategy to reduce Rb metastasis; however, this approach is complicated by the fact that these dormant cells occupy the same BM niche as endogenous hematopoietic stem cells [12]. Increasing evidence suggests that tumor cells do not act alone but are strongly influenced by interactions within a ‘tumor microenvironment’ (TME) [13]. The RB TME is a complex and dynamic ecosystem composed of tumor cells, retinal stromal cells, immune cells, vascular components, and bone marrow-derived mesenchymal stem cells (BM-MSCs) [14]. Therefore, understanding the molecular and cellular mechanisms that support this metastatic environment is essential for improving therapeutic strategies in advanced Rb cases.

BM-MSCs are a class of mesoderm-derived non-hematopoietic stem cells characterized by self-renewal, high proliferation capacity, and multi-directional differentiation potential [15,16]. MSCs are sequestered throughout the adult body in small, stromal niches, a microenvironment that protects and maintains their self-renewal potential and undifferentiated state [17]. MSCs are activated when there is tissue injury, inflammation, or malignancy. They migrate from their ecological niche to the affected site and secrete various cytokines, chemokines, and growth factors that closely interact with the inflammatory environment and the tumor microenvironment, respectively [18,19,20].

Within the TME, BM-MSCs can be reprogrammed into tumor-supportive phenotypes; they secrete growth factors, cytokines, and extracellular matrix components that enhance cancer cell survival, proliferation, invasion, and resistance to therapy [21]. On the other hand, a growing number of studies have demonstrated the beneficial effects of MSC-based therapies in the treatment of various diseases, including ocular disease [22,23,24]. So, the role of MSCs—whether they exert tumor-suppressive or tumor-promoting effects—remains controversial and is particularly poorly defined in the context of Rb.

Small extracellular vesicles (sEVs) have emerged as critical mediators of tumor microenvironment (TME) dynamics by facilitating intercellular communication between tumor cells, stromal cells, and the surrounding microenvironment [25,26]. These vesicles transport tumor-derived oncogenic proteins, lipids, and coding and non-coding RNAs (miRNAs, lncRNAs, and circRNAs), enabling functional reprogramming of target cells, thus promoting tumor survival and progression [27,28]. Tumor-derived EVs have gained considerable interest in the metastasis field because of their ability to promote premetastatic niche formation through the promotion of angiogenesis, immune evasion, extracellular matrix remodeling, and organ-specific colonization [29,30,31]. Numerous in vitro and in vivo studies in Rb and other ocular malignancies have demonstrated that tumor-derived sEVs promote tumor dissemination. They modulate TME by inducing immune suppression, promoting angiogenesis, and enhancing invasive and metastatic potential of the tumor cells [32,33,34,35,36,37,38,39,40,41,42,43,44]. However, the underlying mechanistic role of sEVs in Rb resistance and progression, particularly their effect on cells at metastatic sites such as BM-MSCs, remains largely unexplored.

Therefore, the present study examines functionally the effects of Rb tumor-derived sEVs on cells at the metastatic site, such as BM-MSCs, as well as on tumor cells, using in vitro cellular assays and molecular experiments. These findings provide evidence that Rb-derived sEVs play a role in modulating BM-MSCs and tumor cells to facilitate the establishment of metastasis, by promoting proliferation, migration, differentiation, and enhancing pro-tumorigenic factors and the expression of stemness-associated proteins. The inhibition of sEV-mediated interaction may provide a potential therapeutic strategy in the early stages of metastasis, improve treatment outcomes, and reduce mortality in children with advanced Rb.

## 2. Results

### 2.1. Characterization of sEVs from Tumor and BM-MSC Cell Cultures

**Figure 1:**
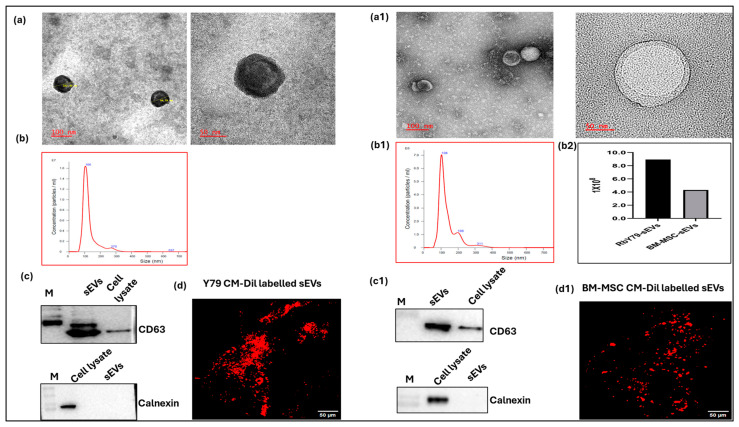
Characterization and labeling of small extracellular vesicles (sEVs) isolated from RbY79 and BM-MSC-conditioned media using SUC followed by the MACS method. (**a**,**a1**) Representative transmission electron microscopy (TEM) images (30,000× magnification) show the morphology of the sEVs. (**b**,**b1**,**b2**) Nanoparticle tracking analysis (Nano Sight) determined the average diameter of the sEVs to be 105.3 ± 50.2 nm (RbY79-derived) and 103.2 ± 56.5 nm (BM-MSC-derived). (**c**,**c1**) Immunoblotting analysis confirmed that sEVs from both sources expressed the surface marker CD63, while the negative marker calnexin was absent. (**d**,**d1**) sEVs from RbY79 and BM-MSC were labeled with CM-DiI dye, with a scale bar of 50 µM and 20× magnification.

To ensure the homogeneity and uniformity of isolated sEVs, we characterized them according to MISEV guidelines. Transmission electron microscopy (TEM; 100 nm and 50 nm) revealed that sEVs isolated from both tumor cells and BM-MSCs displayed a typical round morphology and a size between 30 and 150 nm (Figure 1a,(a1)). Nanoparticle tracking analysis (NTA) showed a vesicle concentration of 8.94 × 10^8^ vesicles/mL for RbY79-derived sEVs, with a mean (mode) size of 128.6 nm (105.3 ± 50.2 nm). In comparison, BM-MSC-derived sEVs exhibited a concentration of 4.31 × 10^8^ vesicles/mL and a mean (mode) size of 136.6 nm (103.2 ± 56.5 nm) (Figure 1b,(b1)). Notably, tumor-derived vesicles showed a higher yield than BM-MSC-derived vesicles (8.94 × 10^8^ vs. 4.31 × 10^8^ vesicles/mL) (Figure 1(b2)). Immunoblotting confirmed the presence of the sEV surface marker CD63 and the absence of calnexin, an ER marker, indicating minimal cellular contamination in the preparations (Figure 1c,(c1)). Additionally, confocal microscopy visualized CM-DiI-labeled sEVs from both tumor cells and BM-MSCs, further confirming successful labeling of isolated sEVs (Figure 1d,(d1)).

### 2.2. Uptake Analysis of Tumor-Derived sEVs by BM-MSC and BM-MSC-Derived sEVs by Tumor Cells

**Figure 2:**
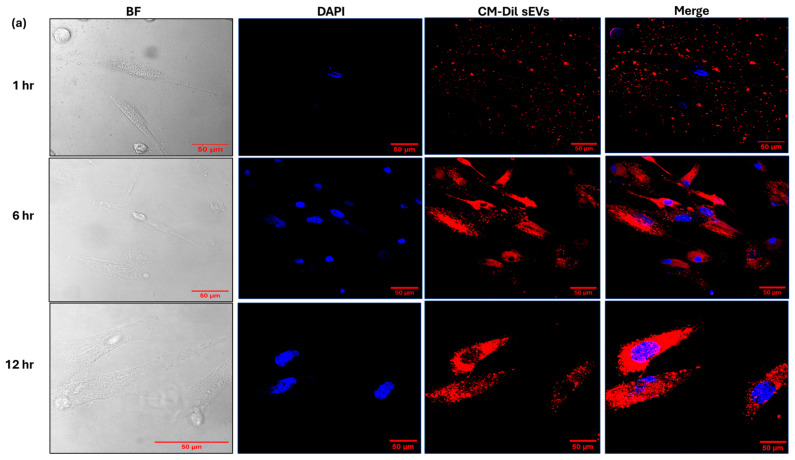

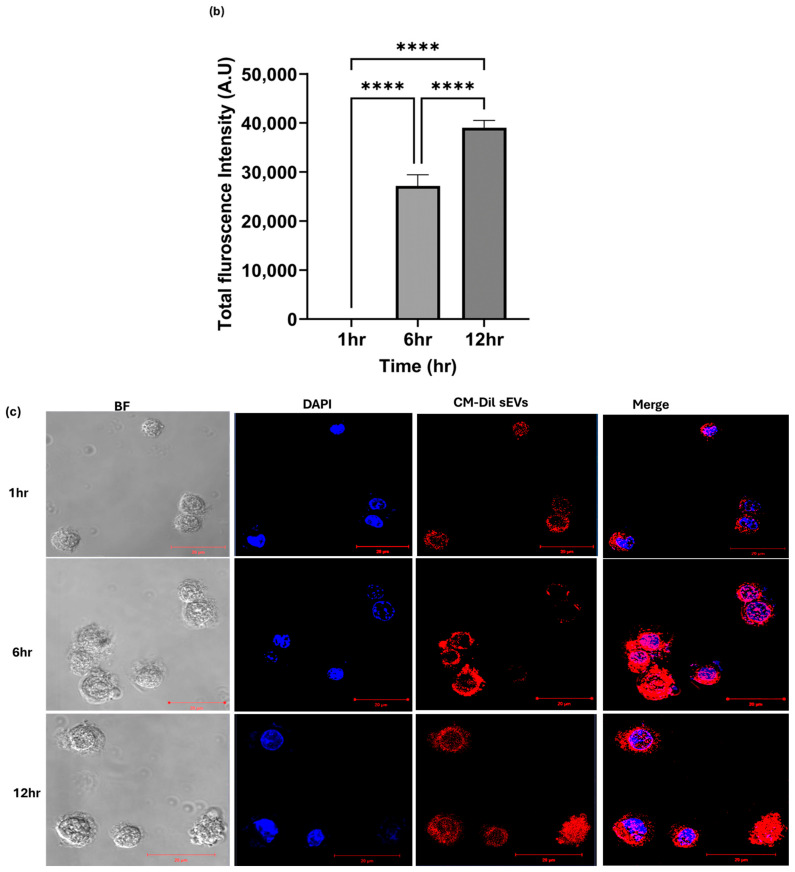

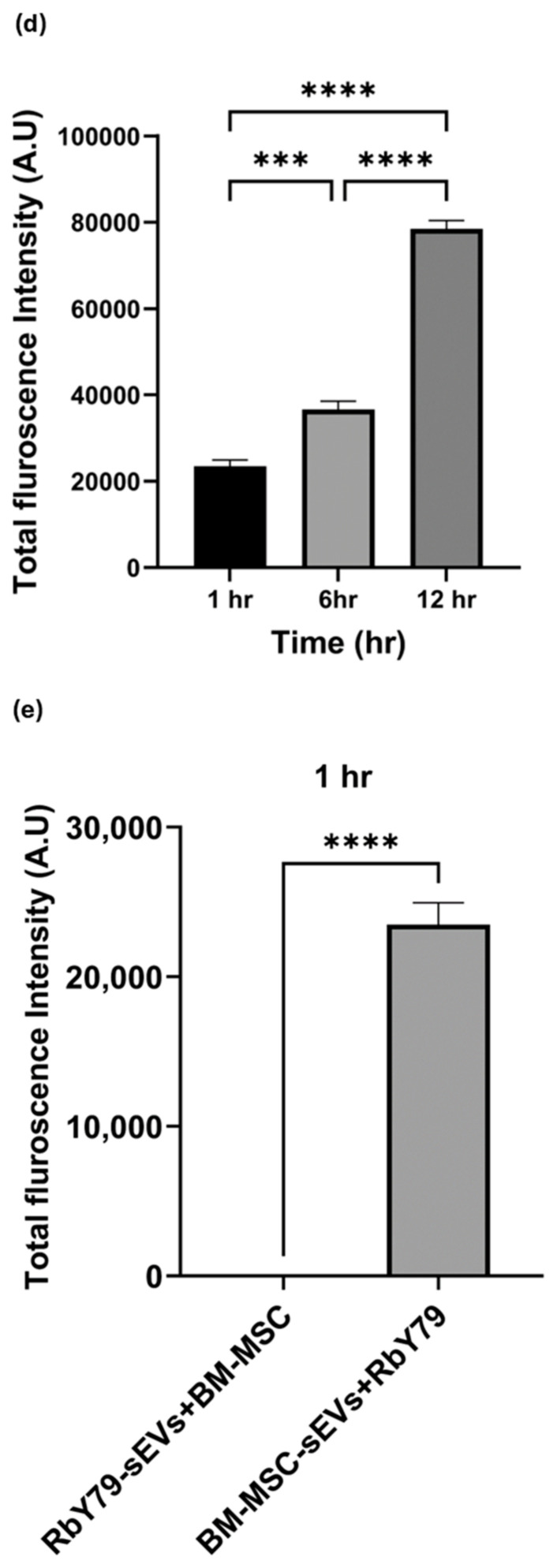
Detection and measurement of labeled sEV uptake by recipient cells (RbY79-sEVs by BM-MSC and BM-MSC-sEVs by RbY79) at different time points of 1 h, 6 h, and 12 h. (**a**) Confocal microscopy images show the uptake of the CM-DiI-labeled sEVs of tumor cells by the BM-MSC cells at 1 h, 6 h, and 12 h. (**b**) Quantitative measurement of fluorescent signal intensity per cell (BM-MSC). Labeled sEVs are visualized in red (CM-DiI), and nuclei are counterstained with DAPI (blue). (**c**) The uptake of the CM-DiI-labeled sEVs of BM-MSCs by RbY79 cells. (**d**) Quantitative analysis of CM-DiI fluorescence intensity per cell (Y79) was performed using Fiji ImageJ 2.9.0/1.54r software to evaluate sEV uptake. (**e**) Quantitative measurement of labeled sEVs in both cells (Y79-derived sEVs on BM-MSC) and (BM-MSC-derived sEVs on Y79) at 1 h. Scale bar: 50 µm, 20 µm. *** *p* < 0.001 and **** *p* < 0.0001 was considered statistically significant, error bars represent the mean ± SEM. N = 3–8.

Fluorescence signal analysis confirmed the presence of RbY79-derived sEVs on the surface of BM-MSCs after 1 h, suggesting relatively slow or minimal uptake during this period. Cytoplasmic fluorescence in BM-MSCs became detectable at 1 h, increased markedly at 6 h, and continued to rise up to 12 h (Figure 2a,b) (*p* < 0.0001). In contrast, BM-MSC-derived sEVs were rapidly internalized by RbY79 tumor cells, with cytoplasmic fluorescence detectable as early as 1 h post-exposure and intensifying significantly between 6 and 12 h, demonstrating efficient uptake (Figure 2c,d). Overall, the BM-MSC-sEVs were internalized by tumor cells more rapidly than the RbY79-sEVs by BM-MSCs, with significant uptake observed within the first hour (Figure 2e) (*p* < 0.0001).

### 2.3. Cell Viability

**Figure 3:**
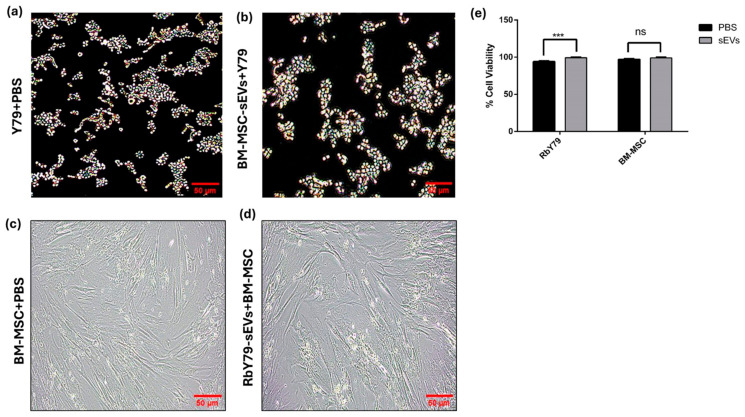
Cell viability of Y79 and BM-MSC cells was assessed using the MTT assay. (**a**,**b**) RbY79 cells were exposed to either PBS or sEVs of BM-MSCs. (**c**,**d**) BM-MSC cells were exposed to either PBS or sEVs of RbY79. (**e**) Quantification of percentage cell viability. *** *p* < 0.001 was considered statistically significant, ns = not significant, error bars represent the mean ± SEM N = 3, scale bar: 50 µm and 20× magnification.

To evaluate the dose-dependent effects of tumor-derived sEVs on BM-MSCs, BM-MSC cells were treated with increasing concentrations of sEVs (0 μg to 50 μg). After 48 h of treatment, the results showed that sEVs derived from Y79 Cells at 30 μg resulted in slightly higher cell viability compared with other concentrations (Supplementary Figure S2). The MTT assay results showed a significant increase in the viability of RbY79 cells following exposure to BM-MSC-derived sEVs compared with RbY79 cells exposed to PBS (Figure 3a,b,e) (*p* < 0.001) at 24 h. In contrast, the viability of BM-MSCs remained unaffected after exposure to RbY79-derived sEVs, and showed no significant difference compared with BM-MSCs exposed to PBS (Figure 3c,d,e). These findings indicate that BM-MSC-derived sEVs exert a tumor-supportive role on RbY79 cells, whereas BM-MSCs are not affected by tumor-derived sEVs within 24 h.

### 2.4. Migration

**Figure 4:**
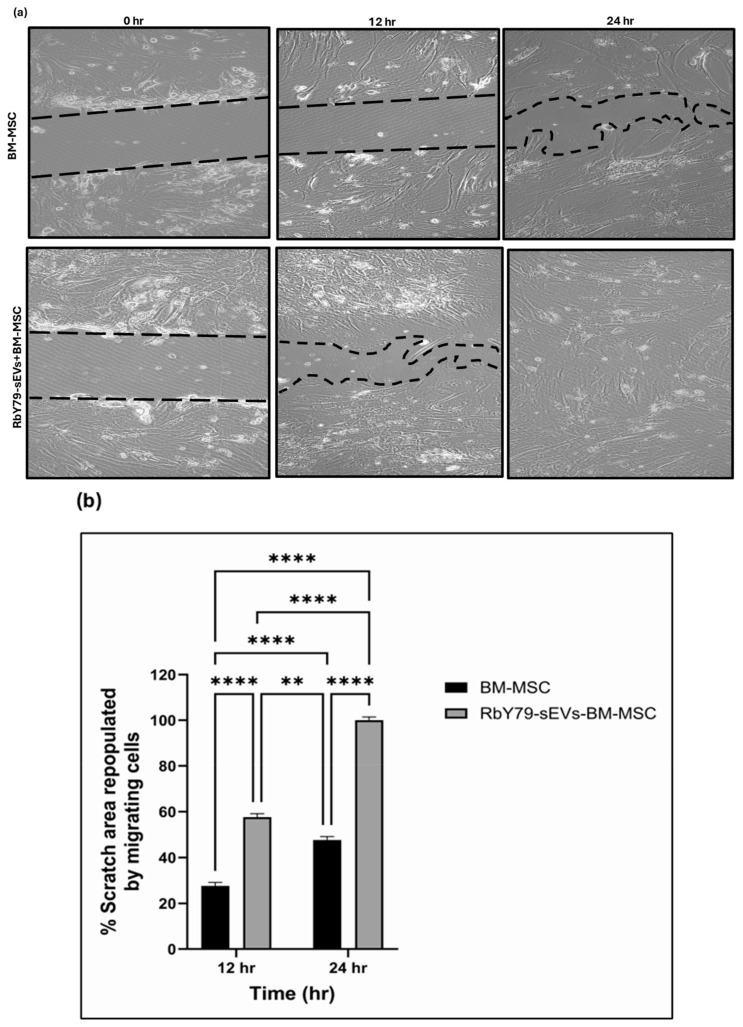

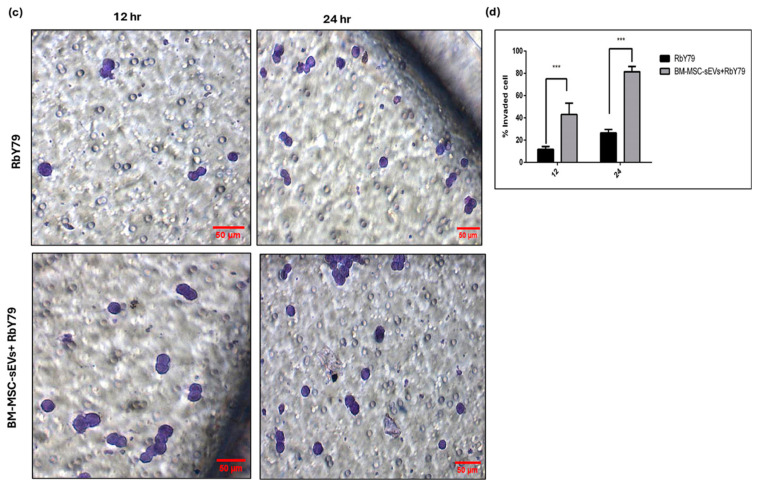
The migration of BM-MSC and RbY79 cell culture after exposure to sEVs. Dashed lines indicate the initial scratch boundaries and wound closure over time. (**a**) Representative images of RbY79-derived sEV exposure to BM-MSC. (**b**) Bar graph illustrating percentage distance closure at indicated time points during the scratch assay. (**c**) Representative images of BM-MSC-derived sEVs exposed to RbY79. (**d**) Bar graph illustrating % of invaded cells at the indicated time points during the Transwell assay. (** *p* < 0.01, *** *p* < 0.001, **** *p* < 0.0001) were considered statistically significant, error bars represent the mean ± SEM. N = 3–8, scale bar: 50 µm and 20× magnification.

The in vitro scratch assay demonstrated a significant acceleration of BM-MSC migration into the cell-free (wound) area in the presence of RbY79-derived sEVs compared with untreated BM-MSCs (Figure 4a,b) (*p* < 0.0001). At 12 h, the scratch area repopulated by migrating cells was approximately 56% for RbY79-sEV-treated BM-MSCs versus 26% for BM-MSCs alone (*p* < 0.0001). At 24 h, the RbY79-sEV-treated BM-MSCs achieved nearly complete closure (~100%), compared with ~45% for the BM-MSCs alone (*p* < 0.0001). These results indicate that BM-MSCs exposed to tumor-derived sEVs demonstrate enhanced migratory capacity over time.

Similarly, the Transwell migration assay showed a significant increase in the percentage of RbY79 cells invading the lower chamber when exposed to BM-MSC-derived sEVs, compared with untreated RbY79 cells (Figure 4c,d) (*p* < 0.0001). At 12 h, approximately 43% of the BM-MSC-sEV-treated RbY79 cells invaded through the membrane, compared with ~12% of the untreated RbY79 cells (*p* < 0.001). At 24 h, invasion increased to ~82% for the BM-MSC-sEV-treated cells versus ~28% for the controls (*p* < 0.001). These results indicate that BM-MSC-derived sEVs strongly enhance the invasiveness of RbY79 cells in vitro.

### 2.5. Phenotypic Changes in BM-MSC Cells

**Figure 5:**
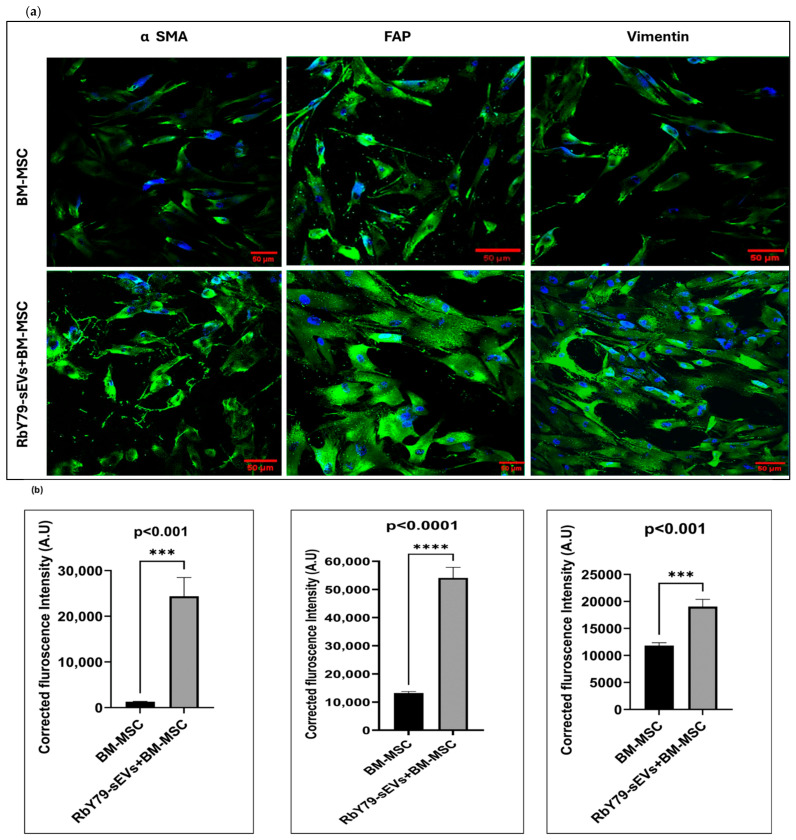
Tumor cell-derived sEVs induce the protein expression of α SMA, FAP, and vimentin in BM-MSCs. (**a**) Immunofluorescence microscopic images for anti-α-SMA, FAP, and vimentin protein expression in BM-MSCs exposed to RbY79-sEVs for 30 days. (**b**) Quantitative fluorescence intensity data showing the mean ± SEM of N = 3–7. *** *p* < 0.001, **** *p* < 0.0001, scale bar: 50 µm and 20× magnification.

Quantitative analysis of the immunofluorescence images from the long-term cultures (30 days) revealed significantly elevated α-SMA expression in RbY79-sEV-treated BM-MSCs when compared to BM-MSCs cultured alone (*p* < 0.001) (Figure 5a,b). Similarly, FAP fluorescence intensity was markedly increased in the RbY79-sEVs + BM-MSC group relative to BM-MSCs alone (*p* < 0.0001). In addition, vimentin expression, as assessed by corrected fluorescence intensity, was significantly higher in RbY79-sEV-treated BM-MSCs compared to the controls (*p* < 0.001), indicating enhanced mesenchymal activation and cytoskeletal remodeling following sEV exposure. Collectively, these findings demonstrate that tumor-derived sEVs significantly promote BM-MSC transdifferentiation, supporting their role in driving fibroblast/myofibroblast activation and stromal reprogramming.

### 2.6. Colony Formation of Tumor Cells upon Exposure to BM-MSCs

**Figure 6:**
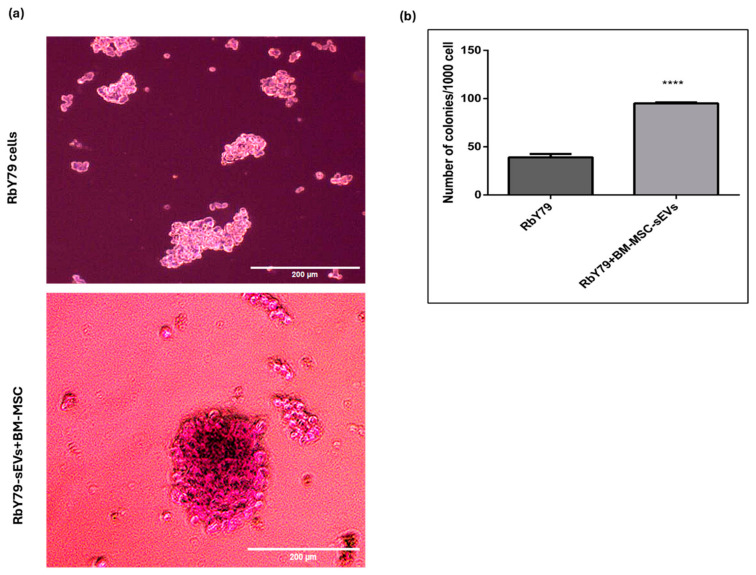
Colony formation of RbY79 cells after exposure to BM-MSC-derived sEVs. (**a**) Untreated RbY79 cells and cells after exposure to BM-MSCs-sEVs. (**b**) Quantitative measurement of the number of colonies of the untreated RbY79 cells and the number after exposure to BM-MSC-derived sEVs. **** *p* < 0.0001 was considered statistically significant, the error bar represents mean ± SEM. N = 3, scale bar: 200 µm and 40× magnification.

Colony formation assays revealed a significant enhancement in the clonogenic potential of tumor cells upon treatment with BM-MSC-derived sEVs. Representative phase-contrast images show an increased number and size of tumor cell colonies in the RbY79 + BM-MSC-sEV group compared with RbY79 cells cultured alone. Quantitative analysis based on cell counting using a hemocytometer after trypan blue staining revealed that untreated RbY79 cells formed an average of 43.4 ± 0.01 colonies. In contrast, exposure to BM-MSC-derived sEVs significantly increased the number of colonies to 97.9 ± 0.001 (*p* < 0.0001) (Figure 6a,b). In addition to increased colony numbers, sEV-treated cultures exhibited more compact spheroid-like colonies, indicating enhanced proliferative capacity and survival. Collectively, these findings suggest that BM-MSC-derived sEVs promote tumor cell clonogenicity and support tumor-growth-associated cellular behavior.

### 2.7. Sphere Formation of Tumor Cells

**Figure 7:**
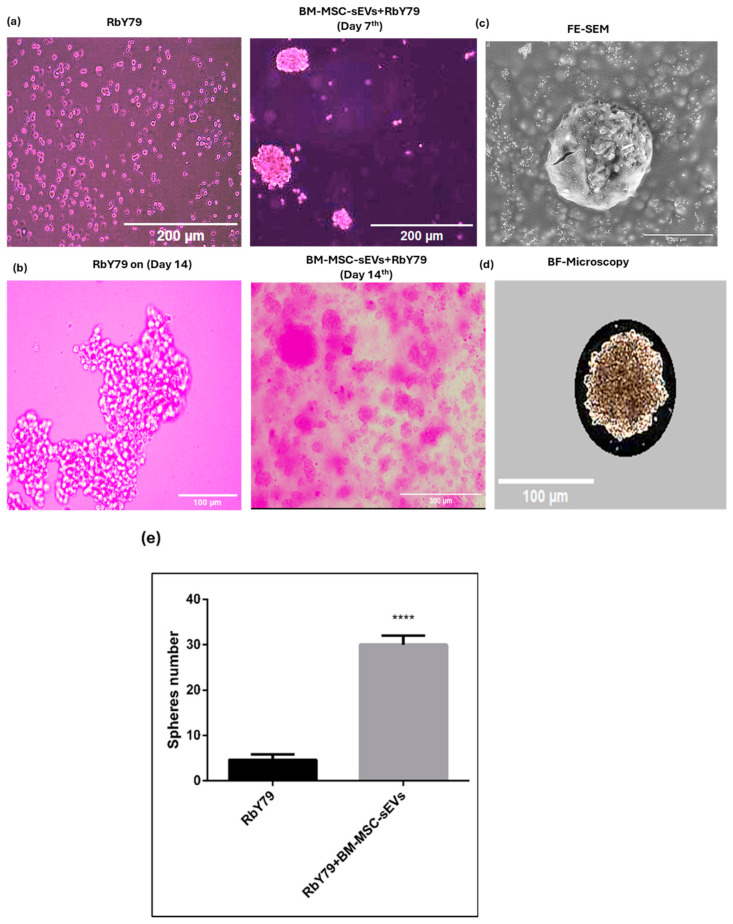
Sphere formation of RbY79 cells after exposure to BM-MSC-sEVs. (**a**) Untreated RbY79 cells and cells after exposure to BM-MSC-derived sEVs up to the 7th day. (**b**) Untreated RbY79 cells and cells after exposure to BM-MSC-derived sEVs after 14 days. (**c**). Field emission scanning electron microscopic image of sphere (FE-SEM) at day 14. (**d**). Bright-field image of sphere at the 14th day (**e**). The ability of RbY79 cells to form spheres increased significantly (**** *p* < 0.0001), error bars represent the mean ± SEM. N = 3–6, scale bar: 200 µm, 100 µm, and 40× magnification.

A hemocytometer was used to quantify the number of spheres formed. RbY79 cells treated with BM-MSC-derived sEVs showed a significant increase in sphere formation from day 7 to day 14 compared to untreated RbY79 control cells (*p* < 0.0001) (Figure 7a,b,e). Field-emission scanning electron microscopy and bright-field imaging further confirmed an increase in sphere size (<100 µm) and compactness in the RbY79 cells (~1 (perimeter^2^(347)/4ᴨx (9578)rea) at 14 days of exposure to BM-MSC-derived sEVs relative to untreated RbY79 cells (Figure 7c,d).

### 2.8. Expression Analysis of Stemness Markers

**Figure 8:**
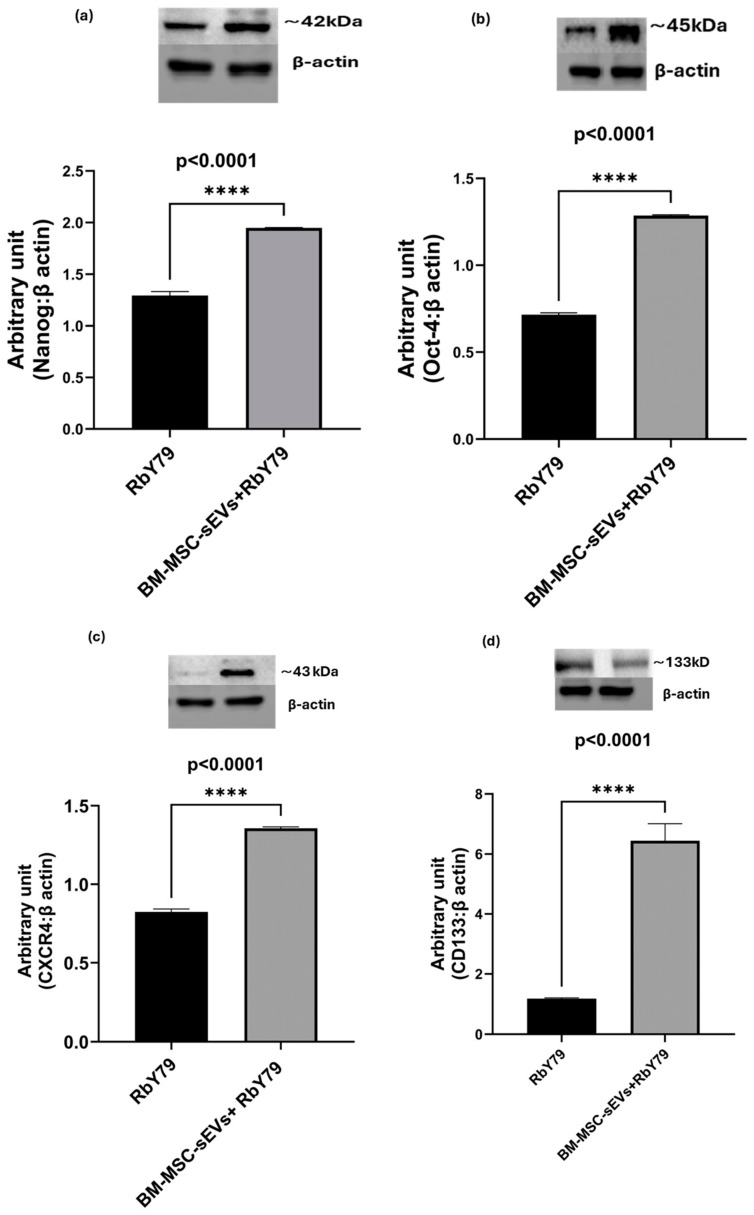
Protein expression of stemness markers in untreated RbY79 and after exposure to BM-MSC-derived sEVs in RbY79 cells. (**a**) NANOG (**b**) OCT-4 (**c**) CXCR4 (**d**) CD133. **** *p* < 0.0001 was considered statistically significant. Error bars represent mean ± SEM. N = 3–5.

As shown in Figure 8a–d, the expression of the stemness markers NANOG, OCT 4, CXCR4, and CD133 was significantly increased in RbY79 cells following exposure to BM-MSC-derived sEVs compared with untreated RbY79 cells (*p* < 0.0001).

### 2.9. Evidence of Altered Cytokine and Chemokine Profile

**Figure 9:**
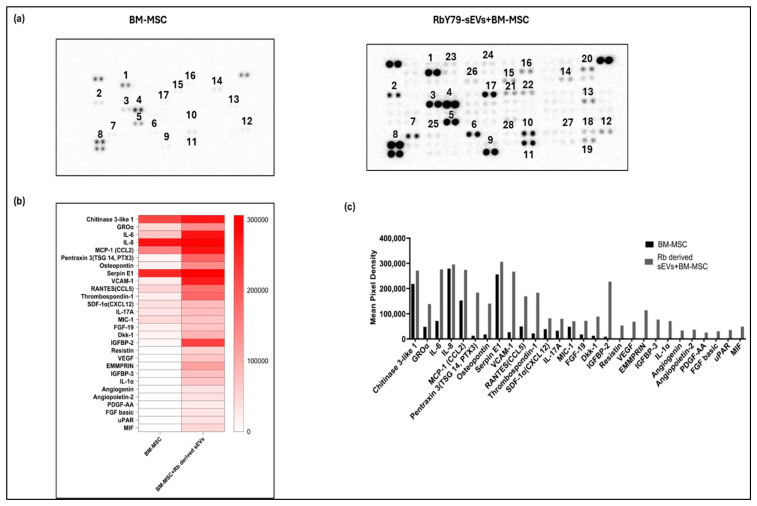

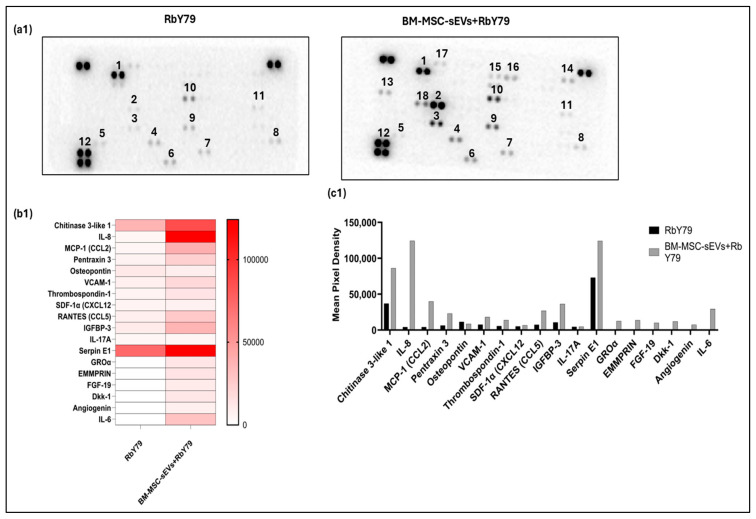
Cytokine and chemokine array blots were incubated with the culture supernatants from both RbY79 and BM-MSC after exposure to sEVs. (**a**) The image represents BM-MSC, which was exposed to tumor-derived sEVs and showed altered protein expression. (**b**) A heat map summarizing the list of differentially expressed proteins. (**c**) Mean pixel density of altered cytokines. (**a1**) The image represents protein expression of RbY79, and RbY79 was exposed to BM-MSC-derived sEVs. (**b1**) Heat map summarizing the list of differentially expressed proteins (**c1**) Mean pixel density of altered proteins between RbY79 and RbY79 cells exposed to BM-MSC-derived sEVs. N = 2.

Cytokine and chemokine profiling revealed that BM-MSCs exposed to RbY79-derived sEVs exhibited a markedly altered secretory profile. Many of the cytokines and chemokines were undetectable in the secretome of the untreated BM-MSCs. Upon exposure to RbY79-sEVs, the BM-MSCs not only secreted several biologically active proteins but also showed increased concentrations in the culture supernatant, including Chitinase 3-like-1, GRO-α, IL-6, IL-8, MCP-1 (CCL2), Pentraxin-3 (PTX-3), Osteopontin, Serpin E1, VCAM-1, RANTES (CCL5), Thrombospondin 1, SDF-1α (CXCL12), IL-17A, MIC-1, FGF-19, Dkk-1, IGFBP-2, Resistin, VEGF, EMMPRIN, IGFBP-3, IL-1α, Angiogenin, Angiopoietin-2, PDGF-AA, FGF basic, uPAR, and MIF (Figure 9a–c). Similarly, RbY79 cells exposed to BM-MSC-derived sEVs displayed enhanced secretion of specific cytokines and chemokines. Proteins that were either absent or present at low levels in untreated RbY79 cells were significantly upregulated following BM-MSC sEV exposure, including Chitinase 3-like-1, IL-8, MCP-1, Pentraxin-3, Osteopontin, VCAM, Thrombospondin 1, SDF-1α (CXCL12), RANTES (CCL5), IGFBP-3, IL-17A, Serpin E1, GRO-α, EMMPRIN, FGF-19, Dkk-1, Angiogenin, and IL-6 (Figure 9(a1–c1)).

## 3. Discussion

The management of Rb metastasis and chemoresistance remains a significant challenge requiring further investigation [10,11,44,45,46,47]. Small extracellular vesicles (sEVs) are increasingly recognized as critical mediators of intercellular communication in the tumor microenvironment [48]. The present study aimed to investigate the role of tumor-derived sEVs (TDsEVs) between metastatic site cells (bone marrow-derived mesenchymal stem cells (BM-MSC) and tumor cells in Rb. This study provides in vitro evidence that tumor-derived sEVs promote a pro-tumorigenic environment by interacting with BM-MSCs that contributes to tumor growth, proliferation, migration, and an enhanced stemness property of tumor (Rb) cells.

In line with MISEV guidelines and previous tumor-derived sEVs studies, our physico molecular characterization results suggested that isolated sEVs displayed the expected size range of 30–150 nm and a characteristic round morphology [49,50]. Their enrichment in CD63 and absence of calnexin confirmed the high purity, homogenety, and integrity of the vesicles, supporting their suitability for subsequent in vitro functional assays [51,52].

Furthermore, fluorescence microscopy enabled the successful labeling of the sEVs, providing a valuable tool for cellular tracking and uptake studies [49,53,54]. A key observation from our study is that the uptake of sEVs by recipient cells is both temporal and cell-type-dependent [55]. Rb-derived sEV uptake by BM-MSCs was delayed, whereas RbY79 cells efficiently internalized MSCs, indicating that sEV uptake occurs in a cell type- and time-dependent manner. This regulated, stepwise internalization is consistent with previous reports demonstrating that sEV uptake kinetics vary among recipient cell types, likely due to various cellular uptake mechanisms such as clathrin-mediated endocytosis, caveolin-mediated endocytosis, macropinocytosis, lipid raft-dependent uptake, and phagocytosis [52,55,56].

Upon uptake of tumor-derived sEVs, BM-MSC cells exert profound functional and phenotypic changes without affecting cell viability [57,58]. Notably, they enhanced BM-MSC migration, indicating that tumor cell-derived sEVs may recruit MSCs to the tumor site [58,59]. The changes in phenotypic marker expression in the BM-MSCs suggest that tumor-derived sEVs reprogrammed the target cells that enhance tumor survival [57]. This kind of transdifferentiation is also observed in other malignancies, including cholangiocarcinoma [59], neuroblastoma [60], colorectal cancer [61], and in gastric cancer [62] highlighting a conserved role for this effect of tumor-derived vesicles on stromal cells [63]. The conversion of MSCs into cancer-associated fibroblasts (CAFs) is a process known to support extracellular matrix remodeling. In addition to phenotypic changes, the reprogrammed BM-MSCs induced a pro-inflammatory and tumor-supportive secretory phenotype. They exhibited upregulation of multiple factors spanning several functional categories. Pro-inflammatory cytokines and chemokines, including IL-6, IL-8, IL-17A, MCP-1 (CCL2), RANTES (CCL5), IL-1α, MIC-1, GRO-α, and Pentraxin-3 (PTX-3), were markedly increased, indicating activation of inflammatory signaling pathways [64]. Elevated angiogenic and vascular factors such as VEGF, Angiogenin, Angiopoietin-2, PDGF-AA, FGF basic, and SDF-1α (CXCL12) suggested altered BM-MSCs promote a pro-angiogenic potential [65,66]. The expression of extracellular matrix modulators and adhesion molecules including Osteopontin, Thrombospondin 1, VCAM-1, EMMPRIN, uPAR, and Serpin E1, suggest a remodeling of the tumor microenvironment that might promote tumor migration. Growth factors and signaling modulators such as FGF-19, Dkk-1, IGFBP-2, IGFBP-3, and Resistin were induced, further supporting MSC reprogramming toward a tumor-promoting phenotype. Other factors, including Chitinase 3-like-1, were also increased, reflecting additional tumor-supportive functions [67,68]. Collectively, this comprehensive shift in BM-MSC signaling demonstrates how tumor-derived vesicles remodel the microenvironment to enhance inflammation, extracellular matrix remodeling, angiogenesis, and metastatic niche formation. In fact, these kinds of tumor-supportive mechanisms are documented in other malignancies as well [59,60,62,69,70,71].

On the other hand, the BM–MSC-derived sEVs exerted pronounced pro-tumorigenic effects upon uptake by tumor cells. BM-MSC-derived sEVs enhanced tumor cell viability and migration, supporting the concept that BM-MSC-derived signals promote tumor aggressiveness [62,72,73]. Furthermore, these sEVs stimulated colony and sphere formation, and upregulated key stemness markers in tumor cells, indicating a phenotypic shift of tumor cells towards a cancer stem cell-like state. This transition may enhance tumor proliferation and self-renewal potential in these cells [63]. This is particularly important given that cancer stemness is closely linked to therapy resistance, metastasis, and tumor relapse [63].

Additionally, BM-MSC-derived sEVs altered the tumor cell secretory profile, inducing the expression of multiple pro-tumorigenic factors associated with tumor growth, angiogenesis, and immune modulation, including Chitinase 3-like-1, IL-8, MCP-1, Pentraxin-3, Osteopontin, VCAM-1, Thrombospondin 1, SDF-1α (CXCL12), RANTES (CCL5), IGFBP-3, IL-17A, Serpin E1, GRO-α, EMMPRIN, FGF-19, Dkk-1, Angiogenin, and IL-6 [74]. These findings suggest that BM-MSC-derived sEV-mediated transfer reprograms tumor cells at both phenotypic and secretory levels, consistent with prior studies demonstrating the role of stem cell-derived sEVs in remodeling the tumor cells to establish a pro-tumorigenic niche that supports metastasis [75,76].

Collectively, these data underscore the ability of tumor-derived sEVs to promote tumor survival, homing, and spread by reprogramming the BM-MSC cells, and highlighting their potential as therapeutic targets in metastatic Rb.

A key limitation of this study is the lack of comprehensive cargo profiling of both tumor- and MSC-derived sEVs, as well as the absence of validation of differentiation markers at the protein and gene expression levels. Moreover, further studies using additional Rb cell lines and in vivo animal models or patient-derived models could provide a more robust understanding that cannot be fully achieved in vitro, which represents a significant gap.

## 4. Materials and Methods

### 4.1. Cell Cultures

The human BM-MSCs used in this study were obtained from our archival source (approved by the IRB), which has been thoroughly characterized and previously published in Investigative Ophthalmology & Visual Science (IOVS) [77]. The retinoblastoma Y79 cells (Riken: RCB1645 Y79) are a generous gift from Dr. S. Krishnakumar, Sankara Nethralaya, Chennai, India. Both cells (BM-MSC and Y79) were revived and cultured in Dulbecco’s modified Eagle medium (1× DMEM, Gibco^TM^ by Thermofisher Scientific, Carlsbad, CA, USA) and Roswell Park Memorial Institute medium (c), respectively, supplemented with 100 U/mL antibiotic–antimycotic (Thermofisher Scientific, MA, USA) and fetal bovine serum (FBS, Gibco^TM,^ Thermofisher Scientific, MA, USA) at 37 °C in a humidified atmosphere in an incubator with 5% CO_2_ until they reached 80% confluency. We selected the Rb-Y79 cell line because it is a well-established and widely used human retinoblastoma model that faithfully recapitulates key molecular and phenotypic features of Rb, making it suitable for proof-of-concept studies. Using a single, well-characterized cell line allowed us to focus on detailed mechanistic analyses with high reproducibility.

### 4.2. Extraction and Characterization of sEVs

Approximately 8 × 10^6^ million cells of RbY79 and 2 × 10^6^ BM-MSC cells were thoroughly washed twice with 1× PBS and cultured in 5 mL of serum-free media. The cellular debris was removed by centrifugation at 300× *g* for 10 min and 2000× *g* for 10 min, followed by centrifugation at 10,000× *g* for 30 min to remove microvesicles. Further, concentrated culture media using 30 kDa centrifugal filters (Amicon^®^ Ultra Centrifugal Filter, 30 kDa MWCO, UFC9030, Merck Millipore, Darmstadt, Germany) and filtered through 0.22 µ syringe filters (PALL Corporation, Acrodisc^R^ Syringe, Port Washington, NY, USA). For sEV extraction, we followed the procedure described previously by Gupta et al. [78]. Approximately 30 mL of concentrated culture media was loaded slowly over 4 mL of 30% sucrose solution (prepared in 1× phosphate-buffered saline or PBS (pH 7.40), forming a layer, and centrifuged at 100,000× *g* and 4 °C for 90 min using a Beckman Coulter (Beckman Coulter, Brea, CA, USA). The supernatant was discarded and the sucrose layer (~5 mL) was resuspended in 1× PBS and ultracentrifuged at 100,000× *g* at 4 °C for 90 min to pellet down the sEVs. After this, the sEVs were resuspended in 1 mL of 1× PBS [78].

The sEVs were further purified using a magnetic-activated cell sorting separator (MACS, Miltenyi Biotec QuadroMACS Separator With MACS Multi Stand) using CD63 according to the manufacturer’s protocol (Miltenyi Biotech, Auburn, AL, USA, cat#130-110-918). The resuspended sEVs were mixed with 50 μL microbeads and vortexed. The mixture was incubated for 1 h at room temperature (RT). A μ-column was placed in the magnetic field of a μMACS separator and equilibrated with 100 μL of equilibration buffer. The magnetically labeled sample was added to the column, followed by rinsing with an isolation buffer. The column was then removed from the magnetic separator and placed into a 1.5 mL tube. Finally, 100 μL of isolation buffer was added to elute the magnetically labeled vesicles, which were collected in the 1.5 mL tube.

### 4.3. Transmission Electron Microscopy (TEM)

The extracted sEVs were diluted 1:100 in PBS, and 5 µL of this suspension was applied onto formvar-coated copper grids for 3 min (200 mesh, Ted Pella Inc, Redding, CA, USA), which were glow-charged for 45 s. Excess liquid was carefully blotted using Whatman filter paper. The grids were then stained with 5 µL of 2% aqueous uranyl acetate for 1 min at room temperature in the dark. After removing the excess stain with Whatman filter paper, the grids were allowed to air-dry overnight at room temperature. Finally, the samples were examined and imaged using a FEI Tecnai G2 S-Twin transmission electron microscope, Hillsboro, OR, USA) operating at an accelerating voltage of 200 kV.

### 4.4. Nanoparticle Tracking Analysis (NTA)

The concentration and size distribution (mean size) of sEVs per volume (mL), were calculated using a NanoSight NS300 (Malvern, England, UK), NTA Version: NTA 3.4. The isolated sEV samples were diluted to 300× (1:300) in PBS for viewing under the camera (Blue laser 405). About 300 µL of the samples was injected into the sample chamber, which captures Brownian motion, and directly viewed with a video camera. The images of the sEVs were captured in video form, and the hydrodynamic diameter of the sEVs was calculated using the Stokes–Einstein equation.

### 4.5. Immunoblotting Analysis

Cells and sEVs lysed with radioimmunoprecipitation assay (RIPA) were buffer-supplemented with a protease inhibitor cocktail. The protein yield of cell lysate and sEVs was measured using a bicinchoninic acid protein assay kit (Biorad, Hercules, CA, USA) according to the manufacturer’s protocol. The protein samples of cell lysate (30 μg) and sEV samples (30 μg) were denatured at 95 °C for 5 min in 4× Laemmli buffer (Life Technologies, Waltham, MA, USA). The denatured proteins were separated by a 10% SDS-PAGE gel and transferred onto nitrocellulose membranes (NC, 0.2µ) (Biorad, Hercules, CA, USA). Blocking with 5% bovine serum albumin (BSA) in 1× PBS incubated overnight was followed by incubating the membranes with the corresponding antibodies CD63, Calnexin (Abcam, Cambridge, MA, USA, 1:1000 dilution), anti-FAP (1:200L; Abcam#ab314456), anti-α-SMA (1:50) (Abcam #AB7817), or anti-vimentin (1:100; Abcam#ab49918). The anti-OCT 4 (1:1000) (Cell Signaling Technology #75463, Danvers, MA, USA), anti-NANOG (1:1000) (Abcam#3580), anti-CD133 (1:1000) (Cell Signaling Technology #51917), and anti-CXCR4 (1:500) antibodies (Ab124824) were obtained from Abcam overnight, at 4 °C, with slow shaking in an orbital shaker. These antibodies were then washed three times with 1× PBS Tween20 (0.05%) buffer and incubated with a goat anti-rabbit IgG HRP-conjugated secondary antibody (Abcam, USA, 1:10,000 dilution) for 2 h at RT. The Femto Chemiluminescence Substrate (G-Biosciences, St. Louis, MO, USA) was used to visualize specific immunoreactive blots and was documented using Chemi doc XRS+ (Bio-Rad Laboratories, Hercules, CA, USA).

### 4.6. Fluorescent Labeling of sEVs by the Incubation Method

To assess and visualize the sEV uptake by Y79 cells and BM-MSC cells, CellTracker CM-Dil 5 μL (0.1 µM from 100 µM, Invitrogen™, Thermo Fisher, Waltham, MA, USA) was added to 30 μg of sEVs, and incubated for 30 min at 37 °C. Excess dye was removed through ultracentrifugation at 150,000× *g* for 90 min at 4 °C. After that, the supernatant was discarded, and the precipitate found at the bottom was resuspended with 1× PBS as previously described [42].

### 4.7. Uptake of sEVs Analyzed Using Confocal Microscopy

After seeding Rb Y79 cells and BM-MSC on the coverslip (5 × 10^3^ cells per well), the cells were maintained in 6-well plates and were exposed to labeled sEVs (30 μg) for 1 h, 6 h, and 12 h. To remove any unbound sEVs, the cells were washed three times with 1× PBS after incubation. Subsequently, the nucleus of the cells was stained with 1 μL DAPI (1:500) (Abcam, USA) at RT for 10 min and rinsed thrice with 1× PBS buffer. The cell suspension of RbY79 (50 μL) and BM-MSC was prepared by mounting 50 μL of 80% glycerol on a glass slide and then covering it with a coverslip. Confocal microscopy (Carl Zeiss, Jena, Germany) was used to track the intracellular concentration of the dye inside the cell.

For live-cell imaging, BM-MSCs (5 × 10^3^ cells) were seeded in a 35 mm dish (cat#81156) containing 1 mL of serum-free medium. Nuclear staining was performed using 1 μL/mL DAPI, and the excess dye was removed by washing three times with 1× PBS. The cells were then focused using phase-contrast optics. Prior to imaging, cells were exposed to RbY79-derived sEVs (30 μg). Time-lapse video recording was carried out between 1 h and 3 h and again at 6 h post-exposure with RbY79-derived sEVs (recorded for 30 min) using Leica TCS SP8 laser scanning confocal microscopy (Leica Microsystems, Wetzlar, Germany), and the images were analyzed by using Fiji Image J software, 2.9.0/1.54r (Supplementary Videos S1 and S2).

### 4.8. Cell Viability Analysis by MTT Assay

Briefly, 5 × 10^3^ cells per well were seeded in a 96-well plate in 100 µL of complete culture medium and allowed to adhere overnight at 37 °C in a humidified incubator with 5% CO_2_. The cell viability by MTT assay was performed on two cell types: (1) Rb Y79 cells, either not exposed or exposed to sEVs derived from BM-MSCs (sEVs, 30 μg) or PBS; and (2) BM-MSCs, evaluated both as not exposed and following exposure to sEVs of RbY79 (30 μg) or PBS for 24 h. Following treatment, 10 µL of MTT solution (5 mg/mL in PBS) (Thermofischer Scientific, Invitrogen^TM^, Waltham, MA, USA) was added to each well and incubated for 3–4 h at 37 °C to allow for the formation of formazan crystals. The medium was carefully removed, and the formazan crystals were dissolved in 100 µL of dimethyl sulfoxide (DMSO). Absorbance was measured at 570 nm using a microplate reader, and cell viability was calculated relative to untreated control cells.

$$\%\;\mathrm{Cell}\;\mathrm{viability}=\frac{{\mathrm{OD}}_{sample}-{\mathrm{OD}}_{blank}}{{\mathrm{OD}}_{Control}-{\mathrm{OD}}_{blank}}\times 100$$

### 4.9. Migration Assay: Scratch and Transwell Migration (Matrigel)

To evaluate BM-MSC migration, both scratch wound healing and Transwell migration assays were performed (Supplementary Figure S1). For the scratch assay, 5.0 × 10^4^ BM-MSCs were seeded in 12-well plates and allowed to adhere for 24 h. Cells were then treated with either PBS (control) or 30 μg of sEVs derived from RbY79 cells at 0 h, 12 h, and 24 h. A uniform scratch was created in the confluent monolayer using a 200-μL pipette tip. Images of the wound area were captured at the indicated time points and analyzed using ImageJ software to quantify wound closure.

To assess the migration of RbY79 cells, a Transwell assay was conducted using 24-well Transwell chambers with 8 μm pore size inserts (Corning, Inc, Corning, NY, USA). The upper chamber was coated with Matrigel (1:5 dilution). A total of 5.0 × 10^4^ cells were seeded into the upper chamber, and 500 μL of serum-free medium was added to the lower chamber in the presence or absence of BM-MSC-derived sEVs. Cells were incubated for 0 h, 12 h, and 24 h. Following incubation, cells that migrated through the membrane were fixed and stained with crystal violet. Migrated cells were visualized under a microscope and quantified by averaging counts from at least five random fields per group using this formula.

$$\%\;\mathrm{Migration}=\frac{{\mathrm{Width}}_{0h}-{\mathrm{Width}}_{t}}{{\mathrm{Width}}_{0h}}\times 100$$

### 4.10. Immunofluorescence

BM-MSC cells were seeded on 1.5 coverslips in 6-well plates and allowed to grow for 30 days, exposed with or without tumor-derived sEVs, which were added every 48–72 h After 30 days, the cells were removed from the plate and fixed with 4% paraformaldehyde in PBS for 10 min at room temperature (RT) and washed three times with 1× PBS. Thereafter, the cells were permeabilized with 0.1% Triton-X 100 (Sigma-Aldrich, St. Louis, MO, USA) for 10 min in RT. After washing with 1× PBS, the coverslips were incubated for 1 h with specific antibodies diluted in 1× PBS containing 5% fetal bovine serum (FBS) at RT. The antibodies used were mouse anti-vimentin (1:200), rabbit anti-FAP (1:200, Abcam#ab314456), and mouse anti-SMA (1:20, Abcam #AB7817). The coverslips were washed twice with 0.05% Tween 20 in 1× PBS for 5 min, and further Nuclei were counterstained with 4′,6-diamidino-2-phenylindole solution in 1× PBS before mounting with DAPI for 10 min at RT (62248, Thermo Fisher Scientific, Waltham, MA, USA) and visualized by confocal fluorescence microscopy (Carl Zeiss, Jena, Germany) with 20× or 40× objective magnification.

### 4.11. Clonogenic Assay

The Rb Y79 cells exposed to BM-MSC-derived sEVs (30 μg) were grown in agarose as single cells to assess their colony-forming potential. A base layer of 0.8% agarose was added to the wells of a 24-well plate, followed by an overlay of the cell suspension (1000 cells/well in 0.48% agarose). The plates were incubated for two weeks, after which the colonies were fixed with 3.7% paraformaldehyde (PFA) and stained with crystal violet. Colony images were captured at 1.5× and 4× magnifications and analyzed using ImageJ and Open CFU software 3.9.0 (http://opencfu.sourceforge.net). To evaluate colony-forming efficiency (CFE), colonies with more than 50 cells were counted, and the percentage of colonies was calculated. Additionally, the colony morphology was analyzed and categorized into holoclones, meroclones, or paraclones.

### 4.12. Spheroid-Formation Assay

RbY79 cells were prepared into single-cell suspensions and centrifuged at 500× *g* for 5 min at RT. Following supernatant removal, the cells were resuspended in a culture medium and counted using a hemocytometer. A total of 5,000 cells per well were seeded into ultralow-adherence 6-well plates (Corning, CLS3471) and incubated for 7 to 14 days with or without BM-MSC-derived sEVs every 3 days. Sphere formation was observed, and the number of spheres in each well was imaged using light microscopy (Olympus CKX53 microscope, Olympus Corporation, Tokyo, Japan).

#### Field Emission Scanning Electron Microscopy (FE-SEM)

To examine the morphology, the spheroids were washed twice with PBS, then fixed using a solution of 4% PFA and 1% glutaraldehyde in PBS for 2 h at RT. Following fixation, the spheroids were dehydrated using ethanol solutions of increasing concentration, spending 15 min in each concentration of 30–100% ethanol. Finally, the spheroids were then coated with carbon, sputtered with gold palladium particles and viewed with an FE-SEM (Zeiss, Jena, Germany).

### 4.13. Cytokine and Chemokine Profiling by Dot Blot Analysis

The secretome (500 μL) was collected from BM-MSC and Rb Y79 cells exposed with or without Rb Y79-sEVs (30 μg) and BM-MSC-sEVs (30 μg) for 48 h. The culture supernatants were carefully collected and first centrifuged to remove cells and debris. The clarified supernatants were then subjected to ultracentrifugation at 150,000× *g* for 90 min to pellet and remove residual extracellular vesicles, including the added sEVs. The resulting sEV-depleted supernatants were subsequently used for the cytokine/chemokine dot blot analysis.

Protein expression was measured following the manufacturer’s protocol (ARY022B, R&D Systems, Minneapolis, MN, USA). Briefly, for blocking, 2 μL of Array Buffer 6 was added to the membranes, which were incubated for 1 h on a shaker. Samples were diluted with Array Buffer 6 (1.5 mL) and added to the membranes, which were incubated overnight at 2–8 °C on a rocking shaker. The membranes were then washed with 1× wash buffer. A detection antibody cocktail (30 μL) diluted in 1.5 mL of 1× array buffer 4/6 was added and incubated for 1 h. After washing, 2 mL of 1× streptavidin-HRP was added and incubated for 30 min at RT. Following a final wash, membranes were dried, and 1 mL of chemi reagent mix was applied evenly to each membrane and incubated for 1 min. Chemiluminescent signals were then captured by exposing the membranes using multiple exposure times for optimal detection.

#### Statistical Analysis

All quantitative data are presented as mean ± standard error of the mean (SEM), and the differences between mean values were evaluated using an unpaired Student’s *t*-test and a two-tailed *t*-test (for two groups). One-way analysis of variance ANOVA followed by Tukey’s post hoc test (for multiple group comparisons) using Prism 10 (GraphPad, Boston, MA, USA) was also performed. The following levels of significance were used to determine statistical significance: ** *p* < 0.01, *** *p* < 0.001, and **** *p* < 0.0001.

## 5. Conclusions

This study demonstrated that tumor-derived small extracellular vesicles (sEVs) mediate interaction between tumor cells and cells (BM-MSCs) of the metastatic site of Rb, promoting the formation of a tumor-supportive milieu. Tumor-derived sEVs induced a cancer-associated fibroblast-like phenotype in BM-MSCs, enhanced their migratory capacity, and altered the expression of pro-inflammatory molecules. Exhibiting bidirectional crosstalk, BM-MSC-derived sEVs are uptaken by tumor cells and in turn promote tumor cell proliferation, stemness, colony, and sphere formation, altering the expression of pro-tumorigenic factors. Collectively, these findings highlight the critical functional role of tumor-derived sEVs in modifying cells of the metastatic site that provide a favorable microenvironment for the establishment of Rb metastases. In future, in vivo studies are warrented to explore the potential of targeting tumor-derived sEV signaling as a novel therapeutic strategy for high-risk Rb patients.

## Data Availability

The original contributions presented in this study are included in the article/Supplementary Materials. Further inquiries can be directed to the corresponding author.

## Supplementary Materials

The following supporting information can be downloaded at https://www.mdpi.com/article/10.3390/ijms27062654/s1.

## Abbreviations

Rb	Retinoblastoma
sEVs	Small Extracellular Vesicles
BM-MSC	Bone Marrow Mesenchymal Stem Cells
GRO-α	Growth-Regulated Oncogene-Alpha
IL-6	Interleukin-6
IL-8	Interleukin-8
MCP-1 (CCL2)	Monocyte Chemoattractant Protein-1 (C-C Motif Chemokine Ligand 2)
αSMA	Alpha Smooth Muscle Actin
FAP	Fibroblast Activation Protein
OCT-4	Octamer-Binding Transcription Factor 4
PROX1	Prospero Homeobox Protein 1
CXCR4	C-X-C Chemokine Receptor 4
NANOG	Homeobox Protein NANOG
VCAM-1	Vascular Cell Adhesion Molecule-1
RANTES (CCL5)	Regulated on Activation, Normal T Cell Expressed and Secreted (C-C Motif Chemokine Ligand 5)
SDF-1α (CXCL12)	Stromal Cell-Derived Factor 1-Alpha (C-X-C Motif Chemokine Ligand 12)
IL-17A	Interleukin-17A
MIC-1	Macrophage Inhibitory Cytokine-1
FGF-19	Fibroblast Growth Factor-19
Dkk-1	Dickkopf-Related Protein 1
IGFBP-2	Insulin-Like Growth Factor Binding Protein-2
VEGF	Vascular Endothelial Growth Factor
EMMPRIN	Extracellular Matrix Metalloproteinase Inducer
IGFBP-3	Insulin-Like Growth Factor Binding Protein-3
IL-1α	Interleukin-1 Alpha
PDGF-AA	Platelet-Derived Growth Factor-AA
FGF basic	Basic Fibroblast Growth Factor
uPAR	Urokinase Plasminogen Activator Receptor
MIF	Macrophage Migration Inhibitory Factor
