# Supplementary material for: Pro-Tumorigenic Signaling Between Small Extracellular Vesicles of Cancer Cells and Bone Marrow-Derived Mesenchymal Stem Cells—An In Vitro Study
Source: Int J Mol Sci. 2026 Mar 13;27(6):2654. doi: 10.3390/ijms27062654 (PMC13026775; doi:10.3390/ijms27062654)
Supplement: Supplementary file 1 [file ijms-27-02654-s001.zip › ijms-4085825-supplementary.pdf]

Pro-Tumorigenic Signaling Between Small Extracellular Vesicles of Cancer Cells and Bone Marrow-Derived Mesenchymal Stem Cells—An In Vitro Study

Jyothi Attem [1], Ram Mukka Raju Jogula [1], Swathi Kaliki [2,3,4] and Geeta K. Vemuganti [1,2,*]

[1] School of Medical Sciences, Science Complex, University of Hyderabad, Hyderabad 500046, India; attemjyothi69@gmail.com or 18bmph01@uohyd.ac.in (J.A.); 17bmph02@uohyd.ac.in or rmraju.in@gmail.com (R.M.R.J.)
[2] L.V Prasad Eye Institute, Hyderabad 500034, India; swathikaliki@lvpei.org
[3] The Operation Eyesight Universal Institute for Eye Cancers, L.V Prasad Eye Institute, Hyderabad 500034, India
[4] Prof. Brien Holden Eye Research Centre, L.V Prasad Eye Institute, Hyderabad 500034, India
* Correspondence: geeta.vemuganti@lvpei.org or gkvemuganti@gmail.com; Tel.:+91-40-68102345; Fax: +91-40-23548271

1. Migration of BM-MSC cells after exposure with RbY79: Tran swell assay

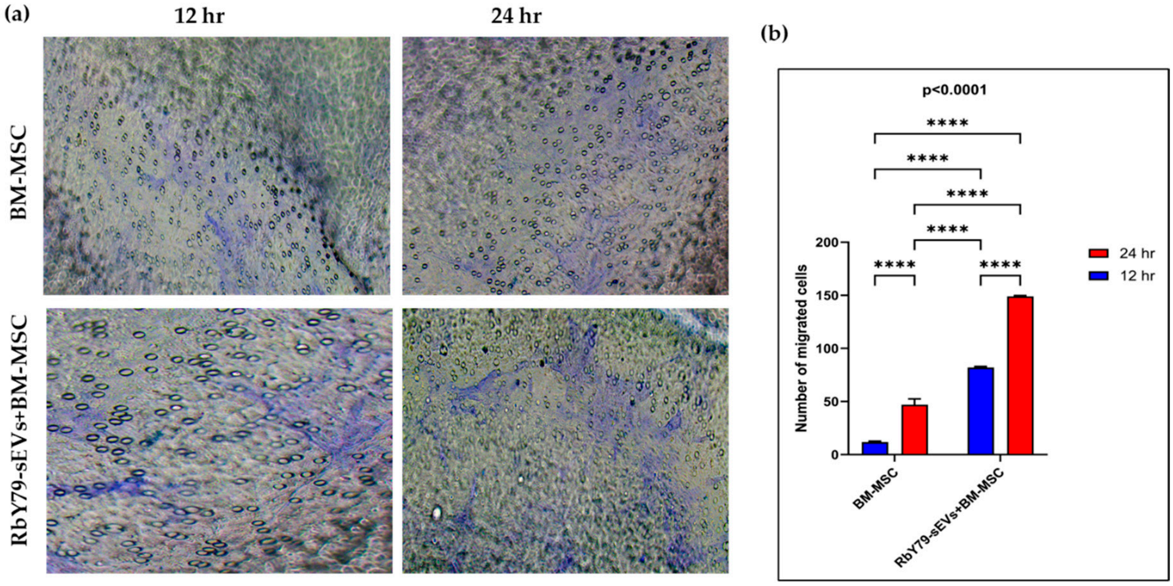

Figure S1. The migration of BM-MSC after exposure to sEVs. (a) Representative images of RbY79-derived sEVs exposure to BM-MSC at 12hr and 24hr. (b) Bar graph illustrating number of migrated cells at the indicated time points during the Transwell assay. (***p<0.001), error bars represent the mean ± SEM of 3 replicates, N=3-8.

2. The effect of Tumor derived sEVs on BM-MSC

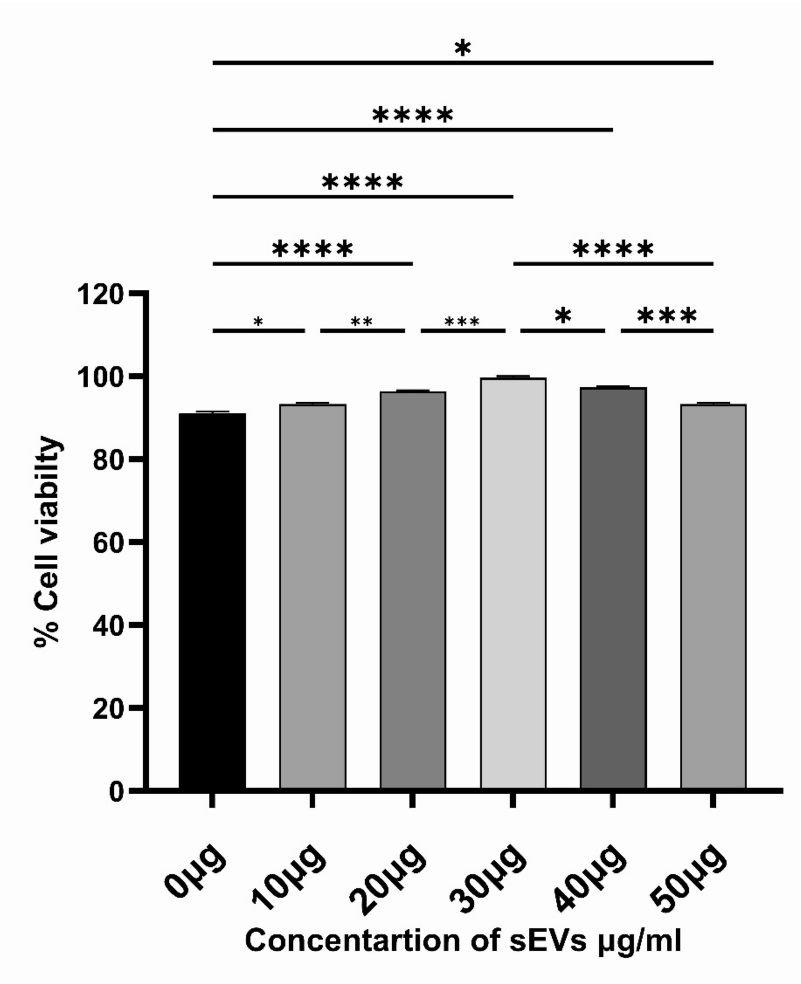

Figure S2. The viability of BM-MSC after exposed with tumor derived sEVs with different concentration.
